# Lycopene Inhibits IL-6 Expression by Upregulating NQO1 and HO-1 via Activation of Nrf2 in Ethanol/Lipopolysaccharide-Stimulated Pancreatic Acinar Cells

**DOI:** 10.3390/antiox11030519

**Published:** 2022-03-08

**Authors:** Jaeeun Lee, Joo Weon Lim, Hyeyoung Kim

**Affiliations:** Department of Food and Nutrition, College of Human Ecology, Yonsei University, Seoul 03722, Korea; jaeeun.chris@gmail.com (J.L.); jwlim11@yonsei.ac.kr (J.W.L.)

**Keywords:** ethanol, lycopene, interleukin-6, nuclear factor erythroid-2-related factor 2, pancreatic acinar cells

## Abstract

In alcoholic pancreatitis, alcohol increases gut permeability, which increases the penetration of endotoxins, such as lipopolysaccharides (LPS). LPS act as clinically significant triggers to increase pancreatic damage in alcoholic pancreatitis. Ethanol or LPS treatment increases reactive oxygen species (ROS) production in pancreatic acinar cells. ROS induce inflammatory cytokine production in pancreatic acinar cells, leading to pancreatic inflammation. The nuclear erythroid-2-related factor 2 (Nrf2) pathway is activated as a cytoprotective response to oxidative stress, and induces the expression of NAD(P)H quinone oxidoreductase 1 (NQO1) and heme oxygenase-1 (HO-1). Lycopene exerts anti-inflammatory and antioxidant effects in various cells. We previously showed that lycopene inhibits NADPH oxidase to reduce ROS and IL-6 levels, and zymogene activation in ethanol or palmitoleic acid-treated pancreatic acinar cells. In this study, we examined whether lycopene inhibits IL-6 expression by activating the Nrf2/NQO1-HO-1 pathway, and reducing intracellular and mitochondrial ROS levels, in ethanol and LPS-treated pancreatic AR42J cells. Lycopene increased the phosphorylated and nuclear-translocated Nrf2 levels by decreasing the amount of Nrf2 sequestered in the cytoplasm via a complex formation with Kelch-like ECH1-associated protein 1 (Keap1). Using exogenous inhibitors targeting Nrf2 and HO-1, we showed that the upregulation of activated Nrf2 and HO-1 results in lycopene-induced suppression of IL-6 expression and ROS production. The consumption of lycopene-rich foods may prevent the development of ethanol and LPS-associated pancreatic inflammation by activating Nrf2-mediated expression of NQO1 and HO-1, thereby decreasing ROS-mediated IL-6 expression in pancreatic acinar cells.

## 1. Introduction

Excessive alcohol consumption is associated with pancreatic damage, including pancreatitis and pathological inflammation of the pancreas. Alcohol-induced oxidative stress is linked to ethanol (EtOH) metabolism in the pancreas [1]. The pancreas metabolizes EtOH via both oxidative and non-oxidative pathways. The oxidative pathway involves the enzymes alcohol dehydrogenase and cytochrome P4502E1 (CYP2E1); these enzymes convert EtOH into acetaldehyde, a toxic metabolite that reacts with proteins and lipids; ultimately leading to cell damage. In the cytosol of acini, CYP2E1 is responsible for approximately 20% of EtOH metabolism at physiological alcohol concentrations [2,3,4]. EtOH-induced increases in reactive oxygen species (ROS) generation and lipid peroxidation have been shown to be blocked by inhibitors of CYP2E1 and anti-CYP2E1 immunoglobulin G [5]. The non-oxidative pathway involves the formation of an ester linkage between EtOH and fatty acids, which is mediated by fatty acid ethyl ester (FAEE) synthases. An increase in FAEE levels has been noted in the pancreas of rats and humans following alcohol consumption [6,7]. Laposata and Lange [8] showed that acetaldehyde could not fully explain alcohol-induced damage in the pancreas, where oxidative metabolism is minimal. Additionally, alcohol intake causes a dose-dependent shift from oxidative to non-oxidative EtOH metabolism, owing to the inhibition of the oxidative pathway [9,10,11]. Although the increase in toxic non-oxidative EtOH metabolites may mainly contribute to pancreatic damage in alcoholic pancreatitis, both oxidative and non-oxidative metabolism of alcohol induce oxidative stress in the pancreas.

In addition, continuous exposure to alcohol disrupts intestinal barrier function by increasing intestinal permeability and the penetration of endotoxins, such as lipopolysaccharides (LPS) [12,13,14]. In a rat model, both acute and chronic alcohol consumption induced damage to the intestinal mucosal membrane by disrupting the intestinal barrier [15]. LPS are structural components of the outer membrane of Gram-negative bacteria. LPS consist of three components: lipid A, a core oligosaccharide, and an O side chain. LPS bind to LPS-binding proteins and are delivered to the cell surface receptor, cluster of differentiation 14 (CD14). LPS are then transferred to the transmembrane toll-like receptor 4 (TLR4) and its accessory protein, myeloid differentiation factor-2 (MD-2). Since CD14 and MD-2 lack a transmembrane domain, TLR4, a crucial receptor for LPS, is necessary to activate the signaling cascade as a second receptor [16,17]. LPS recognition by TLR4 induces oxidative stress and the release of pro-inflammatory cytokines via NF-κB activation in pancreatic acinar cells [18]. Gu et al. [19] showed that alcohol-enhanced acinar cell-specific production of TNFα and IL-6 after LPS injection in alcohol-fed, LPS-injected rats. They also demonstrated that alcohol enhanced LPS-induced TNFα expression, whereas blockade of LPS signaling diminished TNFα production in pancreatic acinar cells in vitro. These studies demonstrate the potential role of LPS in oxidative stress, and the expression of inflammatory cytokines in alcohol-induced pancreatitis.

Mitochondria play a crucial role in the pathogenesis of pancreatitis because ROS are generated during respiratory chain activity [20]. During EtOH metabolism, one molecule of NADH is produced, which produces more reactive components, thereby increasing the activity of the mitochondrial respiratory chain [21]. In mitochondria, NADH is shuttled into respiratory complexes by the malate–aspartate shuttle, the activity of which increases upon EtOH treatment [21]. High concentrations of reducing equivalents facilitate the generation of superoxide anion radicals. These radicals have high oxidative capacity, which accelerates the vicious cycle of ROS production and cell toxicity [22]. In alcoholic pancreatitis, mitochondrial dysfunction (including decreased respiratory rates), lower ATP synthesis, and the impaired ability to import glutathione into mitochondria, have been shown [23], suggesting a possible role of oxidative stress and mitochondrial dysfunction in the pathogenesis of pancreatitis. 

Lycopene, a non-provitamin A carotenoid, is a powerful antioxidant [24,25,26]. It has a variety of biological effects, including anti-inflammatory, anticancer, antidiabetic, cardioprotective, antioxidative, hepatoprotective, and neuroprotective effects [24]. It exhibits a two-fold and ten-fold greater removal of singlet oxygen than beta-carotene and alpha-tocopherol [25]. It can act on ROS, such as hydrogen peroxide, nitrogen dioxide, and hydroxyl radicals [26]. The antioxidant activity of lycopene is mainly dependent on its scavenging properties of singlet oxygen and hydroxyl radicals. Lycopene reportedly decreased the expression of inducible nitric oxide synthase and TNF-α level in the pancreatic tissues of rats with L-arginine-induced pancreatitis [27]. We previously showed that lycopene reduced ROS levels, and inhibited nuclear factor-κB (NF-κB) activation and the expression of IL-6, in cerulein-stimulated pancreatic acinar cells [28]. The supplementation of antioxidants, including lycopene, reduced the levels of IL-6 in older women [29], and the intake of lycopene reduced the serum levels of oxidative stress indicators, such as lipid peroxides [30]. Thus, dietary supplementation of carotenoids may be beneficial for preventing the development of pancreatitis, especially in individuals that are malnourished due to alcohol consumption. The consumption of naturally occurring lycopene-rich fruits and vegetables, including tomatoes, watermelon, pink grapefruit, pink guava, and papaya, is recommended for the prevention of oxidative stress-associated inflammatory diseases, including pancreatitis [31]. The inflammatory cytokine IL-6 is a highly suitable prognostic marker for severe pancreatitis, as IL-6 levels correlate with disease severity in both experimental and human pancreatitis [32,33,34]. Therefore, in this study, we aimed to determine the effect of lycopene on IL-6 expression to investigate whether lycopene supplementation is beneficial in preventing alcohol/LPS-induced pancreatitis by activating antioxidant mechanisms.

Recently, we demonstrated that lycopene inhibits EtOH/palmitoleic acid-induced mitochondrial dysfunction, zymogen activation, and IL-6 expression, by reducing NADPH oxidase-mediated ROS production in pancreatic acinar cells [35]. Morris-Stiff et al. [36] found that patients with chronic pancreatitis had significantly lower plasma concentrations of antioxidants (selenium, vitamin A, vitamin E, β-carotene, xanthine, β-cryptoxanthin, and lycopene) than control subjects. A systematic review demonstrated that low-grade inflammation is reduced by lycopene supplementation in humans [37]. These authors suggested that it is beneficial to occasionally consume lycopene-rich foods in order to stay healthy and maintain the circulation of lycopene at basal levels.

Lycopene supplementation (50 mg/kg BW/day) has been shown to decrease neuronal oxidative damage by increasing nuclear factor erythroid-2-related factor 2 (Nrf2) activity and increasing the expression of Nrf2-targeted genes NAD(P)H, quinone oxidoreductase 1 (NQO1) and heme oxygenase-1 (HO-1), as a cytoprotective response to oxidative stress in H2O2-treated SH-SY5Y cells [38]. As alcohol/LPS increases the levels of ROS and inflammatory cytokines, reducing ROS levels using lycopene may be beneficial for preventing the development of acute pancreatitis. This prompted us to investigate whether lycopene activates Nrf2 to induce the expression of the antioxidant enzymes NQO1 and HO-1 in pancreatic acinar cells stimulated with EtOH/LPS.

Nrf2, a Cap ‘n’ Collar (CNC)-basic leucine zipper (bZIP) transcription factor, is responsible for antioxidant stress responses and drug detoxification in mammals [39]. The activation of Nrf2 is associated with many inflammatory diseases, as Nrf2 blocks inflammation by directly inhibiting inflammatory cytokine transcription and NF-κB activity [40]. Nrf2 is normally suppressed in the cytoplasm by binding to Kelch-like ECH-associated protein 1 (Keap1). Keap1 promotes ubiquitination and constantly degrades Nrf2 under normal cellular conditions. Under stressful conditions, Nrf2 dissociates from the Nrf2–Keap1 complex and translocates into the nucleus, where it binds to the ARE sequence, leading to the activation of ARE-mediated gene expression [41,42,43]. The phosphorylation of Nrf2 promotes the disruption of the Nrf2–Keap1 complex [44]. Nrf2 dimerizes with small Maf proteins and binds DNA as an obligatory heterodimer to induce the transcription of antioxidant enzymes, such as NQO1 and HO-1 [45,46].

NQO1, a FAD-dependent flavoprotein, is an obligate two-electron reductase of quinones, quinone imines, and nitroaromatics, and it converts quinones into their corresponding hydroquinones using NADH or NADPH as hydride donors [47,48]. NQO1 can, therefore, prevent quinone electrophiles from participating in either sulfhydryl depletion or one-electron reduction, thereby reducing the generation of semiquinones and reactive oxygen intermediates during redox cycling. NQO1 also exhibits superoxide scavenging activity [49].

Heme oxygenase-1 (HO-1) is the rate-limiting enzyme of the heme degradation process, which, in addition to removing toxic heme, produces carbon monoxide, free iron, and biliverdin. Biliverdin is then converted by biliverdin reductase to bilirubin, which serves as an endogenous radical scavenger [50]. HO-1 inhibits NADPH oxidase p47phox and p67phox subunit activity, thus decreasing ROS generation and preventing oxidative damage [51]. Collectively, the Nrf2-target genes NQO1 and HO-1 may protect against pancreatic inflammation by suppressing oxidative stress-mediated cytokine expression in pancreatic acinar cells.

The current study aimed to investigate the inhibitory effects and mechanisms of lycopene on EtOH/LPS-induced oxidative stress and IL-6 expression in pancreatic acinar AR42J cells. To determine the possible antioxidant mechanism of lycopene, we examined the effect of lycopene on Nrf2 activation, and the induction of NQO1 and HO-1, in AR42J cells stimulated with EtOH/LPS. 

## 2. Materials and Methods

### 2.1. Cell Line and Culture Conditions

The rat pancreatic acinar cell line AR42J (pancreatoma; ATCC CRL 1492) was obtained from American Type Culture Collection (Manassas, VA, USA) and cultured as described previously [28]. 

### 2.2. Experimental Protocol

To determine the effect of lycopene (L9879, Sigma-Aldrich), dissolved in tetrahydrofuran (THF), on Nrf2 activation to induce the expression of NQO1 and HO-1, AR42J cells (1.0 × 10^6^/10 mL/dish) were treated with lycopene (at a final concentration of 0.5 μM) for 1, 2, and 3 h, and the expression levels of phosphorylated and total Nrf2, Keap1, NQO1, and HO-1 were assessed in whole-cell extracts or nuclear extracts. AR42J cells incubated with THF (0.01%) alone served as a vehicle control. For each experiment, lycopene-untreated cells received a vehicle THF (0.01%) alone instead of lycopene. 

To assess the dissociation of the Nrf2–Keap1 complex, cells (1.0 × 10^6^/10 mL/dish) were treated with lycopene (at a final concentration of 0.5 μM) for 1 h, and the interaction between Nrf2 and Keap1 was determined in whole-cell extracts and whole-cell extract-derived immunoprecipitates, obtained using the anti-Nrf2 and anti-Keap1 antibodies by precipitation. To observe the nuclear translocation of Nrf2, AR42J cells (2 × 10^5^/2 mL/well in 6-well plates) were treated with lycopene (at a final concentration of 0.5 μM) for 1 h, and confocal microscopic images of immunofluorescence staining of the fixed cells were obtained.

To determine the appropriate incubation time for ROS and IL-6 mRNA expression, time course experiments were performed after treatment of AR42J cells with EtOH/LPS. AR42J cells (2.0 × 10^5^/2 mL/well in 6-well plates) were treated with EtOH (at a final concentration of 250 mM)/LPS (10 μg/mL) for 15, 30, 45, and 60 min (for intracellular and mitochondrial ROS levels) or 1, 2, 4, and 6 h (for IL-6 mRNA expression). AR42J cells were pretreated with lycopene (at a final concentration of 0.1, 0.2, or 0.5 μM) for 1 h, and then treated with EtOH (at a final concentration of 250 mM)/LPS (10 μg/mL) for 30 min (to measure intracellular and mitochondrial ROS levels, mitochondrial membrane potential (MMP), and ATP levels), 6 h (to determine IL-6 mRNA expression), or 24 h (to measure IL-6 protein levels).

To assess the involvement of Nrf2, NQO1, and HO-1 in the inhibitory effect of lycopene on EtOH/LPS-induced IL-6 expression, the cells were pretreated with the Nrf2 inhibitor ML385 (at a final concentration of 5 μM, SML1833-5MG, Sigma, St. Louis, MO, USA) or HO-1 inhibitor ZnPP (zinc protoporphyrin, at a final concentration of 1 μM, sc-691550, Santa Cruz, CA, USA) in the presence of lycopene (at a final concentration of 0.5 μM) for 1 h before EtOH/LPS stimulation. ML385 and ZnPP were dissolved in dimethyl sulfoxide (DMSO). For each experiment, ML385 or ZnPP-untreated cells received a vehicle DMSO (0.05%) alone instead of ML385 or ZnPP.

### 2.3. Preparation of Whole-Cell and Nuclear Extracts

Whole-cell extracts were prepared by the method described previously [52]. Nuclear extracts were prepared using a NE-PER^®^ nuclear extraction kit (Thermo Fisher, Waltham, MA, USA). Nuclear extract specificity was confirmed by the level of lamin B1. Protein concentrations were determined using the Bradford assay (Bio-Rad Laboratories, Hercules, CA, USA).

### 2.4. Measurement of Intracellular ROS Levels

Intracellular ROS levels were determined using dichlorofluorescein diacetate (DCF-DA; Sigma-Aldrich). The cells were incubated with 10 µM DCF-DA in 5% CO_2_/95% air at 37 °C for 30 min. Subsequently, the medium was removed and the cells were washed with phosphate-buffered saline (PBS). The intensities of 2′,7′-dichlorofluorescein (DCF) fluorescence in the cells (in 6-well plates) were measured at 522 nm (excitation at 498 nm) with a Victor 5 multilabel counter (PerkinElmer Life and Analytical Sciences, Boston, MA, USA). Intracellular ROS levels were normalized to the cell number and expressed as the relative percentage of control cells.

### 2.5. Measurement of Mitochondrial ROS Levels

The levels of mitochondrial ROS were measured by MitoSOX (Life Technologies, Grand Island, NY, USA). The cells were incubated with 10 µM MitoSOX in 5% CO_2_/95% air at 37 °C for 30 min. Following this, the cells were washed and scraped into PBS. The intensity of MitoSOX fluorescence at 585 nm (excitation at 524 nm) was measured using a Victor 5 multilabel counter (PerkinElmer Life and Analytical Sciences). Mitochondrial ROS levels were normalized to the cell number and expressed as a relative percentage of controls.

### 2.6. Assessment of MMP

MMP was assessed using 5,5,6,6-tetrachloro-1,1,3,3-tetraethyl benzimidazolyl carbocyanine iodide (JC-1) reagent (1:100; 10009908, Cayman Chemical Company, Ann Arbor, MI, USA). After removing the media, the cells were dried for 15 min at 20–22 °C, washed twice with PBS for 5 min, and mounted with mounting solution (M-7534, Sigma-Aldrich, St. Louis, MO, USA). JC-1 fluorescence (red, excitation at 590 nm and emission at 610 nm; green, excitation at 485 nm and emission at 535 nm) was measured with a confocal laser scanning microscope (LSM 880, Carl Zeiss Inc., Oberkochen, Germany). Fluorescent images were used in conjunction with NIH ImageJ 5.0 software (National Institutes of Health, Bethesda, MD, USA) to determine the relative ratio of red/green fluorescence intensities. MMP level in control cells (cells not treated with EtOH/LPS, lycopene, ML385, or ZnPP) was set as 100%. Data are expressed as the mean ± SE (*n* = 3 in each group).

### 2.7. Measurement of ATP Levels

ATP levels were measured using a luminescent ATP detection assay kit, according to the manufacturer’s protocol (ab113849; Abcam, Cambridge, UK). This ATP detection assay kit is used to measure the level of ATP within the cell. The luminescent ATP assay involves lysis of the cell sample, addition of luciferase enzyme and luciferin, and measurement of the emitted light using a microplate-based luminometer. Cells (2.0 × 10^5^ cells/2 mL/well) in 6-well plates were pretreated with lycopene and subsequently stimulated with EtOH/LPS for 30 min. Following this, a substrate buffer was added to the lyophilized ATP substrate to prepare a luminescent substrate solution. The luminescence was measured using a Victor 5 multilabel counter. The ATP concentration (μM) was determined by interpolating within the ATP standard reference. ATP level in control cells (cells treated without EtOH/LPS, lycopene, ML385, or ZnPP) was set as 100%.

### 2.8. Western Blotting

Western blot analysis was performed using a previously described method [53]. Briefly, the whole-cell or nuclear extracts (20–50 μg protein/lane) were separated by 8–12% SDS polyacrylamide gel electrophoresis and transferred onto nitrocellulose membranes by electroblotting. The proteins on membranes were blocked and incubated with specific antibodies against Nrf2 (ab62352; Abcam, Cambridge, UK), p-Nrf2 (ab76026, Abcam), HO-1 (ADI-SPA-895, Enzo Life Science Inc., Farmingdale, NY, USA), Keap1 (8047S, Cell Signaling Technology, Danvers, MA, USA), NQO1 (ab2346, Abcam), lamin B1 (ab16048, Abcam), and actin (sc-1615, Santa Cruz Biotechnology, Dallas, TX, USA), diluted in TBS-T containing 3% non-fat dry milk overnight at 4 °C, followed by incubation with secondary antibodies (anti-goat, anti-mouse, or anti-rabbit conjugated to horseradish peroxidase from Santa Cruz Biotechnology). Proteins were visualized using Clarity Western ECL Substrate (705061; Bio-Rad). 

Protein levels were determined by densitometry analysis of whole-cell extracts or nuclear extracts, using actin as the loading control and lamin B1 as the index for the nuclear extracts. The ratios of Keap1/actin, p-Nrf2/Nrf2, HO-1/actin, and NQO1/actin in whole-cell extracts, and Nrf2/lamin B1 in nuclear extracts, represent mean ± S.E. from three immunoblots. The ratio at 0 h or that of control cells (cells without EtOH/LPS stimulation and without any treatment) was set at 100%.

### 2.9. Real-Time PCR Analysis for IL-6

Total RNA was isolated using TRI Reagent^®^ (Molecular Research Center, Inc., Cincinnati, OH, USA) and reverse-transcribed into cDNA using a random hexamer and MuLV reverse transcriptase (Promega, Madison, WI, USA). cDNA was used for real-time PCR with primers specific for IL-6 and β-actin. The sequences of the IL-6 primers used to produce the desired 590 bp PCR products were 5-GAGAGGAGACTTCACAGAGGATACCA-3 (forward primer) and 5-CCACAGTGAGGAATGTCCACAA-3 (reverse primer). For β-actin cDNA production, a 349-bp PCR product was obtained using the forward primer 5-ACCAACTGGGACGACATGGAG-3 and reverse primer 5-GTGAGGATCTTCATGAGGTAGTC-3′. The thermal cycling conditions were as follows: 35 cycles of denaturation at 95 °C for 30 s, annealing at 53 °C for 30 s, and extension at 72 °C for 45 s. During the first cycle, the 95 °C step was extended for 3 min. The β-actin gene was amplified in the same reaction to serve as the reference gene.

### 2.10. Enzyme-Linked Immunosorbent Assay (ELISA) for IL-6

The level of IL-6 in the medium was determined using an ELISA kit (R&D Systems, Minneapolis, MN, USA), according to the manufacturer’s instructions.

### 2.11. Immunoprecipitation of the Nrf2–KEAP1 Complex

Immunoprecipitation of the Nrf2–KEAP1 complex was determined by using the method described previously [54]. Briefly, the cells were lysed in immunoprecipitation buffer and centrifuged. Polyclonal antibody and protein G-agarose were added to the supernatant, and the mixture was incubated overnight. The protein G-antibody–antigen complex was collected with immunoprecipitation buffer. The pellet was resuspended in SDS sample buffer and boiled. The preparations were subjected to western blot analysis.

### 2.12. Immunofluorescence Staining

Immunofluorescence staining was assessed by the method described previously [54]. Briefly, The cells were fixed, permeabilized, and incubated with a primary antibody against Nrf2. And then, the cells were incubated with a rhodamine-conjugated mouse anti-rabbit IgG antibody (sc-2492, Santa Cruz Biotechnology). After removal of the secondary antibody, the cells were washed and coverslipped with Vectashield antifade medium containing 40,6-diamidino-2-phenylindole (DAPI). Cells stained with rhodamine-conjugated antibodies were examined under a confocal laser scanning microscope (Zeiss LSM 880, Carl Zeiss Inc., Thornwood, NY, USA). For measuring theNrf2 red fluorescence in the nuclei, ZEN Blue 3.1 software (Carl Zeiss Inc., Thornwood, NY, USA) was used. 

### 2.13. Statistical Analysis

One-way analysis of variance followed by Tukey’s post hoc test was used for statistical analysis. All values are expressed as the mean ± SE (*n* = 12 in each group). Statistical significance was set at *p* ≤ 0.05.

## 3. Results

### 3.1. Lycopene Induces Activation of Nrf2 and Expression of Nrf2-Target Genes NQO1 and HO-1 but Decreases the Interaction between Keap1 and Nrf2 in AR42J Cells

**Figure 1:**
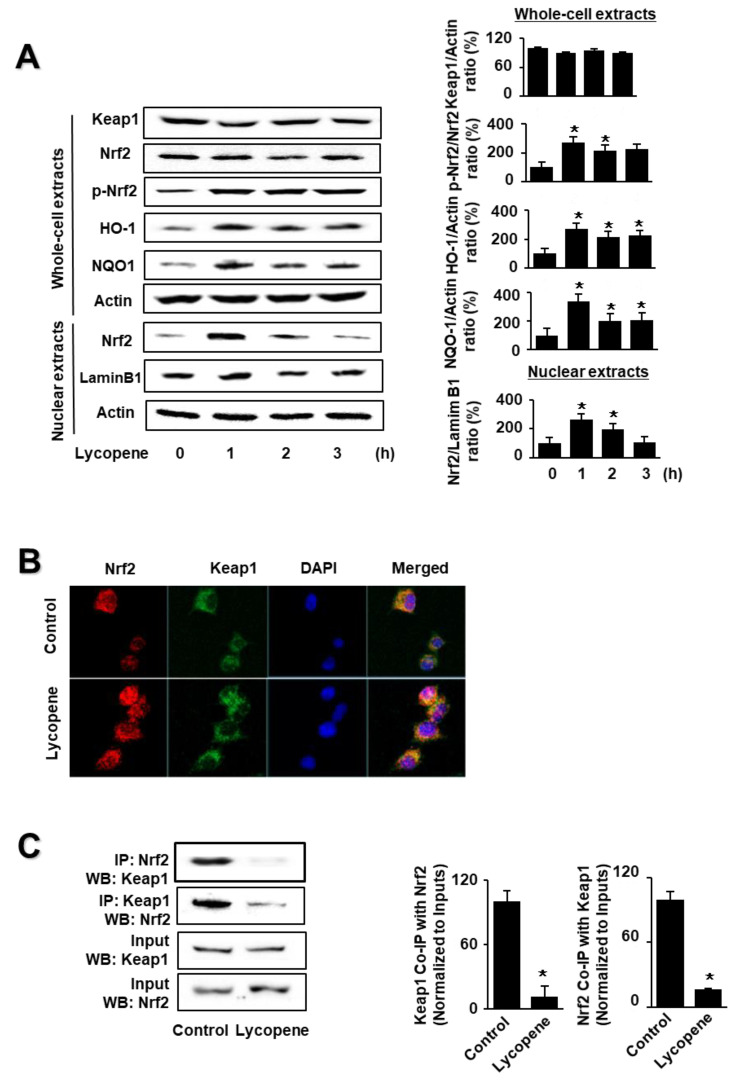
Effect of lycopene on the levels of nuclear factor erythroid-2-related factor 2 (Nrf2), p-Nrf2, Kelch-like ECH-associated protein 1 (Keap1), heme oxygenase-1 (HO-1), NAD(P)H quinone oxidoreductase 1 (NQO1), and Keap1-bound Nrf2 in AR42J cells. Cells (1 × 10^6^/10 mL/dish) were treated with lycopene for the indicated periods (**A**). Cells (1 × 10^6^/10 mL/dish) were treated with lycopene for 1 h (**B**,**C**). (**A**) Protein levels were determined by western blot analysis of whole-cell extracts or nuclear extracts, using actin as the loading control and lamin B1 as the index for the nuclear extracts (**left panel**). The densitometry analysis-derived ratios of Keap1/actin, p-Nrf2/Nrf2, HO-1/actin, and NQO1/actin in whole-cell extracts, and Nrf2/lamin B1 in nuclear extracts, represent mean ± S.E. from three immunoblots. The ratio at the start of the experiment (at 0 h) was set at 100% (**right panel**). (**B**) Immunofluorescence was performed to observe the nuclear translocation of Nrf2 using confocal microscopic images of AR42J cells treated with lycopene (0.5 μM) for 1 h. Nrf2 was visualized using fluorescein/rhodamine-conjugated anti-rabbit IgG antibody (red) with DAPI counterstaining (blue) of the same field. (**C**) Interaction between Nrf2 and Keap1 was determined via immunoprecipitation–western blot (IP-WB) analysis of lycopene-treated cells immunoprecipitated with anti-Nrf2 and anti-Keap1 antibodies (**left panel**). The densitometry analysis-derived ratios of Keap1 Co-IP with Nrf2/Input and Nrf2 Co-IP with Keap1/Input represent mean ± S.E. from three immunoblots. The ratio of control cells (cells without lycopene treatment) was set at 100% (**right panel**). * *p* < 0.05 vs. control. The cells incubated with THF (0.01%) alone served as a vehicle control. For each experiment, control cells received a vehicle THF (0.01%) alone instead of lycopene.

As shown in Figure 1A, lycopene increased the levels of phosphorylated Nrf2, HO-1, and NQO1 in whole-cell extracts, as well as Nrf2 levels in nuclear extracts. Nuclear Nrf2 levels, and the levels of NQO1 and HO-1, peaked at 1 h and subsequently decreased until the termination of lycopene treatment at 3 h. The levels of actin, the loading control, and lamin B1 (the index for nuclear extracts) were not altered by lycopene treatment.

Following this, we investigated the effect of lycopene on the nuclear translocation of Nrf2. AR42J cells were incubated with lycopene for 1 h and subjected to immunofluorescence staining. The level of DAPI, a nuclear marker, was unchanged; conversely, the nuclear level of Nrf2, as determined by red immunofluorescence staining, increased (Figure 1B). These results show that lycopene increased the nuclear translocation of Nrf2 in AR42J cells.

As the activity of Nrf2 is inhibited by Keap1 through protein–protein interactions, we examined whether lycopene changes the interaction between Keap1 and Nrf2 in AR42J cells. We performed coupled immunoprecipitation and western blot analysis using anti-Nrf2 and anti-Keap1 antibodies. Cells (2 × 10^5^/2 mL/well) were treated with or without lycopene for 1 h. The protein levels of Keap1 and Nrf2 in the whole-cell extracts of lycopene-treated cells were unchanged (Figure 1C, lower panel, Input), whereas those in the immunoprecipitated fractions significantly decreased (Figure 1C, upper pane, IP). These results suggest that lycopene disturbs the interaction between Keap1 and Nrf2, indicating that lycopene increases Nrf2 activity by inhibiting the Keap1-mediated sequestration of Nrf2.

### 3.2. EtOH/LPS Increases the Levels of Intracellular and Mitochondrial ROS and IL-6 mRNA in AR42J Cells

**Figure 2:**
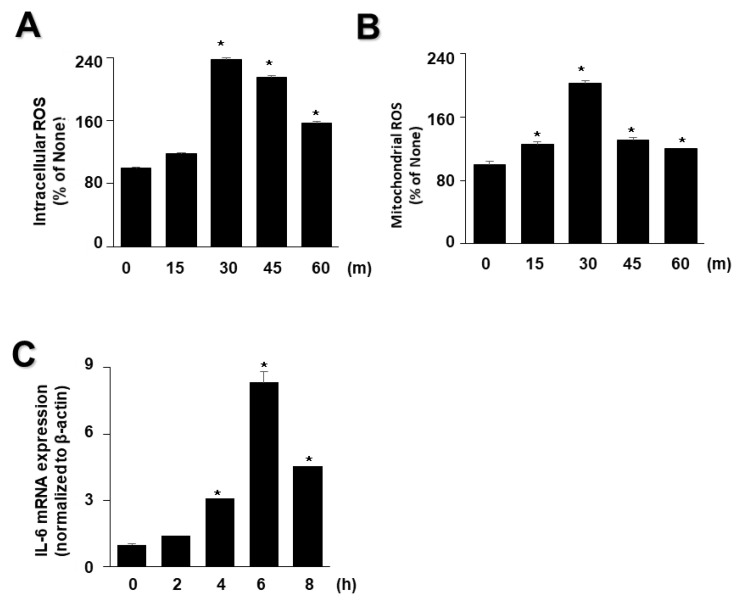
Effect of ethanol (EtOH)/lipopolysaccharides (LPS) on the levels of reactive oxygen species (ROS) and IL-6 mRNA in AR42J cells. Cells (2 × 10^5^/2 mL/well) were stimulated with EtOH (at a final concentration of 250 mM)/LPS (10 μg/mL) for the indicated periods. (**A**) Intracellular ROS levels were determined by dichlorofluorescein (DCF) fluorescence. (**B**) Mitochondrial ROS levels were measured by determining the level of fluorescent MitoSOX. The value for ROS levels at the start of the experiment (at 0 min) was set at 100%. (**C**) mRNA expression of IL-6 was determined by real-time polymerase chain reaction and normalized to that of β-actin. The IL-6 mRNA level at the start of the experiment (at 0 h) was set as 1. Data are expressed as the mean ± SE (*n* = 12 in each group). * *p* < 0.05 vs. the cells at the start of the experiment (at 0 min or 0 h).

As high levels of inflammatory cytokines and ROS are crucial factors in the progression of acute pancreatitis, we examined whether EtOH/LPS increases the levels of IL-6 and ROS in AR42J cells using time course experiments. As shown in Figure 2, EtOH/LPS increased intracellular and mitochondrial levels of ROS and IL-6 mRNA in AR42J cells; intracellular and mitochondrial ROS levels were maximum at 3 min, and then tended to decrease until 60 min (Figure 2A,B), whereas IL-6 mRNA levels peaked at 6 h, and then decreased (Figure 2C). Thus, for further studies on the effect of lycopene on EtOH/LPS-induced increases in IL-6 mRNA and ROS levels, cells were pretreated with lycopene for 1 h, followed by treatment with EtOH/LPS for 30 min (for ROS levels) or 6 h (for IL-6 mRNA expression levels).

### 3.3. Lycopene Inhibits EtOH/LPS-Induced Increase in ROS and IL-6 Levels by Activating Nrf2 and Inducing the Expression of NQO1 and HO-1 in AR42J Cells

**Figure 3:**
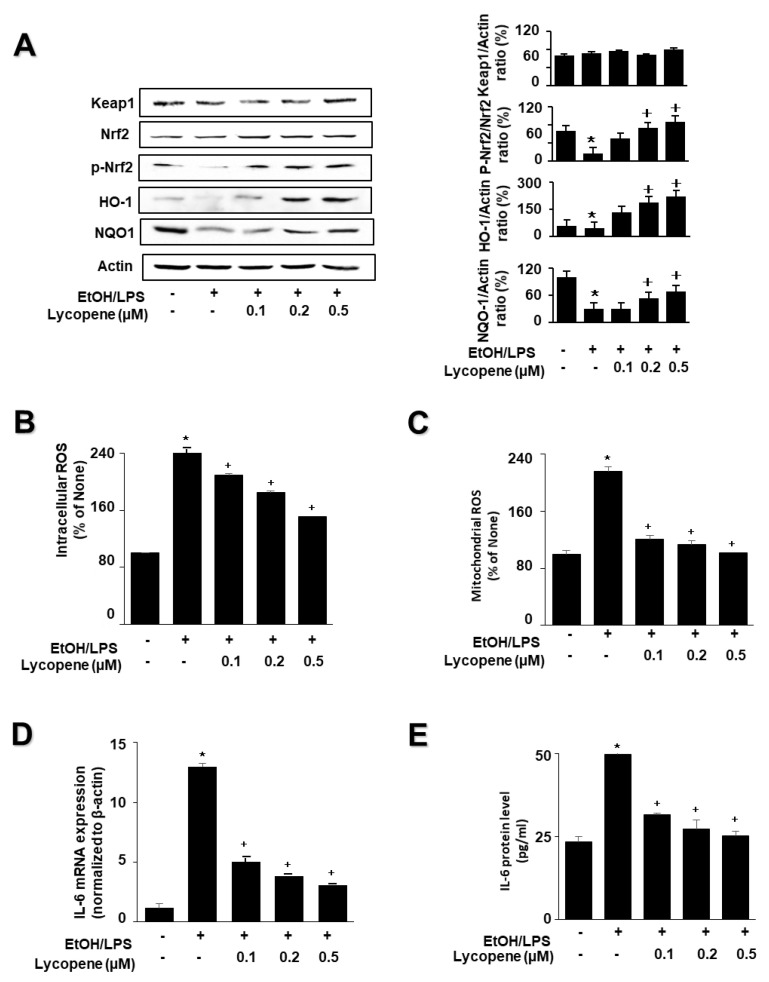
Effect of lycopene on ethanol (EtOH)/lipopolysaccharides (LPS)-induced changes in the levels of phosphorylated Nrf2, HO-1, NQO1, ROS, and IL-6 in AR42J cells. Cells (1 × 10^6^/10 mL/dish or 2 × 10^5^/2 mL/well) were pretreated with the indicated concentrations of lycopene for 1 h, and subsequently stimulated with EtOH (at a final concentration of 250 mM)/LPS (10 μg/mL) for 30 min. (**A**) Protein levels were determined by western blot analysis of whole-cell extracts, using actin as the loading control (**left panel**). The densitometry analysis-derived ratios of Keap1/actin, p-Nrf2/Nrf2, HO-1/actin, and NQO1/actin in whole-cell extracts represent mean ± S.E. from three immunoblots. The ratio of control cells (cells without EtOH/LPS stimulation and without any treatment) was set at 100% (**right panel**). (**B**) Intracellular ROS levels were determined by DCF fluorescence. (**C**) Mitochondrial ROS levels were measured by determining the level of fluorescent MitoSOX. The value for ROS levels in control cells (cells in the absence of EtOH/LPS and without lycopene treatment) was set at 100%. (**D**) mRNA expression level of IL-6 was determined by real-time polymerase chain reaction and normalized to that of β-actin. The IL-6 mRNA level in control cells (cells in the absence of EtOH/LPS and without lycopene treatment) was set as 1. (**E**) Levels of IL-6 in the medium were measured using enzyme-linked immunosorbent assay. Data are expressed as the mean ± SE (*n* = 12 in each group). * *p* < 0.05 vs. control cells (cells without EtOH/LPS stimulation and without lycopene treatment). The cells incubated with tetrahydrofuran (THF, 0.01%) alone served as a vehicle control. For each experiment, lycopene-untreated cells received a vehicle THF (0.01%) alone instead of lycopene. +, treatment.

To investigate the effect of lycopene on the expression of HO-1 and NQO1 in EtOH/LPS-stimulated AR42J cells, cells (1.0 × 10^6^/10 mL/dish) were pretreated with lycopene for 1 h, and then stimulated with EtOH/LPS for 30 min. EtOH/LPS treatment decreased the levels of phosphorylated Nrf2, HO-1, and NQO1 in AR42J cells. Lycopene inhibited these alterations caused by EtOH/LPS (Figure 3A). The levels of Keap1 and total Nrf2 were unchanged following EtOH/LPS treatment with or without lycopene.

To investigate the effect of lycopene on ROS levels and IL-6 expression, the cells were pretreated with lycopene (0.1, 0.2, or 0.5 μM) for 1 h, and then stimulated with EtOH/LPS for 30 min (for ROS levels, Figure 3B,C), 6 h (for IL-6 mRNA expression, Figure 3D), or 24 h (for IL-6 protein levels in the medium, Figure 3E). Lycopene suppressed the EtOH/LPS-induced increase in intracellular and mitochondrial ROS, and IL-6 mRNA and protein levels.

**Figure 4:**
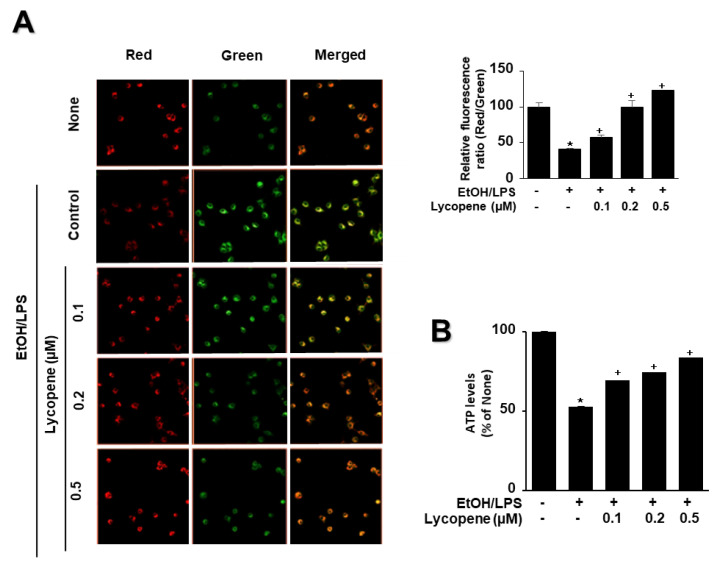
Effect of lycopene on ethanol (EtOH)/lipopolysaccharides (LPS)-induced decrease in mitochondrial membrane potential (MMP) and ATP levels in AR42J cells. Cells (2 × 10^5^/2 mL/well) were pretreated with the indicated concentrations of lycopene for 1 h, and subsequently stimulated with EtOH (at a final concentration of 250 mM)/LPS (10 μg/mL) for 30 min. (**A**) The cells were stained with JC-1 dye and visualized with a confocal laser scanning microscope (left panel). The MMP was determined by measuring the intensity of red emission relative to the intensity of green emission (right panel). A decrease in the red/green fluorescence intensity ratio indicates mitochondrial depolarization. Data are expressed as the mean ± SE (*n* = 3 in each group). (**B**) ATP levels were quantified using luminescent ATP substrate. The ATP concentration was determined by interpolating within the ATP standard reference. The fluorescence ratio or ATP level in control cells (cells not treated with EtOH/LPS or lycopene) was set as 100%. Data are expressed as the mean ± SE (*n* = 12 in each group). * *p* < 0.05 vs. control cells (cells not treated with EtOH/LPS and lycopene); + *p* < 0.05 vs. stimulated cells without lycopene (cells treated with EtOH/LPS and without lycopene). AR42J cells incubated with tetrahydrofuran (THF, 0.01%) alone served as a vehicle control. For each experiment, lycopene-untreated cells received a vehicle THF (0.01%) alone instead of lycopene. +, treatment.

To determine whether EtOH/LPS induces mitochondrial dysfunction in AR42J cells, their MMPs were measured by cytofluorimetric analysis using a JC-1 fluorescent probe. The formation of red fluorescent J-aggregates leads to a membrane potential-sensitive color shift. The green and red fluorescence images (Figure 4A, left panel), and the ratios of red to green fluorescence (Figure 4A, right panel), are here reported to show relative MMP. EtOH/LPS treatment decreased MMP, as validated by the observed decrease in the red/green fluorescence ratio (Figure 4A, right panel; “cells not treated with EtOH/LPS and lycopene” vs. “cells treated with EtOH/LPS but not lycopene”). Lycopene suppressed the EtOH/LPS-induced decrease in the ratio of red/green fluorescence, which indicates that lycopene inhibits EtOH/LPS-induced decrease in MMP in a dose-dependent manner (Figure 4A, right panel; “cells treated with EtOH/LPS but not lycopene” vs. “cells treated with EtOH/LPS and lycopene”). ATP levels decreased after EtOH/LPS treatment, and pretreatment with lycopene blocked this decrease in a dose-dependent manner (Figure 4B).

### 3.4. Nrf2 Inhibitor ML385 Blocked the Effect of Lycopene on the Levels of HO-1, NQO1, ROS, and IL-6 in EtOH/LPS-Stimulated AR42J Cells

**Figure 5:**
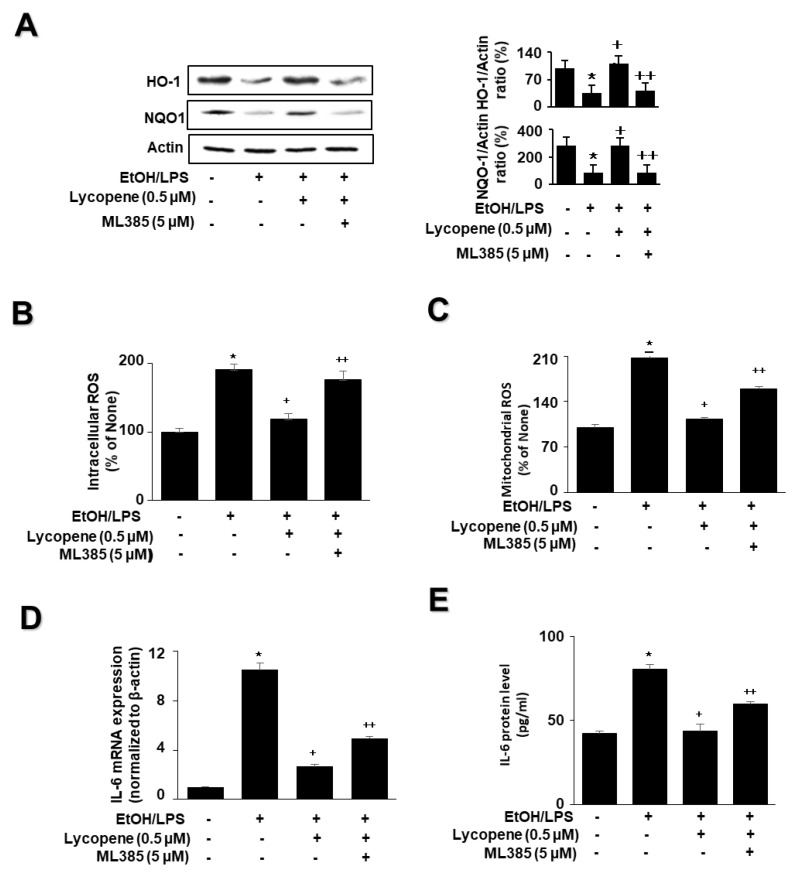
Effect of the Nrf2 inhibitor ML385 on the levels of HO-1, NQO1, ROS, and IL-6 in ethanol (EtOH)/lipopolysaccharide (LPS)-stimulated and lycopene-treated AR42J cells. The cells (1 × 10^6^/10 mL/dish or 2 *×* 10^5^/2 mL/well) were pretreated with ML385 (5 μM) in the presence of lycopene (0.5 μM) for 1 h and stimulated with EtOH (at a final concentration of 250 mM)/LPS (10 μg/mL) for 30 min (**A**), 6 h (**B**), or 24 h (**C**). (**A**) Protein levels of NQO1 and HO-1 in whole-cell extracts were determined by western blot analysis, with actin as the loading control (**left panel**). The densitometry analysis-derived ratios of HO-1/actin and NQO1/actin in whole-cell extracts represent mean ± S.E. from three immunoblots. The ratio of control cells (cells not treated with EtOH/LPS or lycopene) was set at 100% (**right panel**). (**B**) Intracellular ROS levels were determined by DCF fluorescence. (**C**) Mitochondrial ROS levels were measured by determining the level of fluorescent MitoSOX. The value for ROS levels in control cells (cells not treated with EtOH/LPS, lycopene, or ML387) was set at 100%. (**D**) mRNA expression of IL-6 was determined by real-time polymerase chain reaction and normalized to that of β-actin. The IL-6 mRNA level in control cells (cells not treated with EtOH/LPS, lycopene, or ZnPP) was set as 1. (**E**) IL-6 levels in the medium were measured by enzyme-linked immunosorbent assay. Data are expressed as the mean ± SE (*n* = 12 in each group). * *p* < 0.05 vs. control cells (cells not treated with EtOH/LPS, lycopene, or ML387); + *p* < 0.05 vs. cells with EtOH/LPS stimulation and without any treatment; ++ *p* < 0.05 vs. cells with EtOH/LPS stimulation and lycopene treatment. The cells incubated with tetrahydrofuran (THF, 0.01%) alone served as a vehicle control. For each experiment, lycopene-untreated cells received a vehicle THF (0.01%) alone instead of lycopene. ML385 was dissolved in dimethyl sulfoxide (DMSO). For each experiment, ML385-untreated cells received a vehicle DMSO (0.05%). +, treatment.

To determine whether Nrf2 is involved in the effect of lycopene on the levels of HO-1, NQO1, ROS, and IL-6 in EtOH/LPS-stimulated AR42J cells, the cells were pretreated with the Nrf2 inhibitor ML385 (5 µM) in the presence of lycopene for 1 h, followed by EtOH/LPS stimulation for 30 min (for the levels of NQO1, HO-1, and ROS), 6 h (for IL-6 mRNA), or 24 h (for IL-6 protein). ML385 suppressed the inhibitory effect of lycopene on the EtOH/LPS-induced reduction in NQO1 and HO-1 levels (Figure 5A). ML385 reversed the inhibitory effect of lycopene on intracellular ROS (Figure 5B), mitochondrial ROS (Figure 5C), IL-6 mRNA (Figure 5D), and IL-6 protein levels (Figure 5E) in EtOH/LPS-stimulated AR42J cells. Taken together, ML385 reversed the effects of lycopene on EtOH/LPS-induced changes in the levels of NQO1, HO-1, ROS, and IL-6 in EtOH/LPS-stimulated AR42J cells.

### 3.5. A HO-1 Inhibitor ZnPP Blocks the Effect of Lycopene on the Levels of ROS and IL-6 in EtOH/LPS-Stimulated AR42J Cells

**Figure 6:**
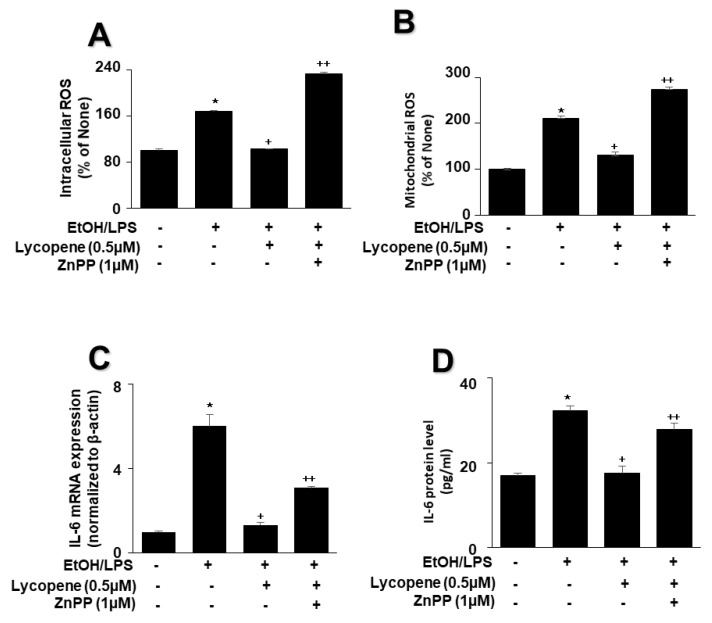
Effect of the HO-1 inhibitor ZnPP on the levels of ROS and IL-6 in ethanol (EtOH)/lipopolysaccharide (LPS)-stimulated and lycopene-treated AR42J cells. Cells (2 × 10^5^/2 mL/well) were pretreated with ZnPP (1 μM) in the presence of lycopene (0.5 μM) for 1 h and stimulated with EtOH (at a final concentration of 250 mM)/LPS (10 μg/mL) for 30 min (**A**,**B**), 6 h (**C**), or 24 h (**D**). (**A**) Intracellular ROS levels were determined by DCF fluorescence. (**B**) Mitochondrial ROS levels were measured by determining the level of fluorescent MitoSOX. The value for ROS levels in control cells (cells not treated with EtOH/LPS, lycopene, or ZnPP) was set at 100%. (**C**) mRNA expression of IL-6 was determined using real-time polymerase chain reaction and normalized to that of β-actin. The IL-6 mRNA level in control cells (cells not treated with EtOH/LPS, lycopene, or ZnPP) was set as 1. (**D**) Levels of IL-6 in the culture medium were measured by enzyme-linked immunosorbent assay. Data are expressed as the mean ± SE (*n* = 12 in each group). * *p* < 0.05 vs. control cells (cells not treated with EtOH/LPS, lycopene, or ZnPP); + *p* < 0.05 vs. cells with EtOH/LPS stimulation and without any treatment;. ++ *p* < 0.05 vs. cells with EtOH/LPS stimulation and lycopene treatment. The cells incubated with tetrahydrofuran (THF, 0.01%) alone served as a vehicle control. For each experiment, lycopene-untreated cells received a vehicle THF (0.01%) alone instead of lycopene. ZnPP was dissolved in dimethyl sulfoxide (DMSO). For each experiment, ZnPP-untreated cells received a vehicle DMSO (0.05%). +, treatment.

To evaluate the involvement of HO-1 in the effect of lycopene on EtOH/LPS-stimulated ROS and IL-6 levels, cells were pretreated with the HO-1 inhibitor ZnPP (1 µM) in the presence of lycopene for 1 h, followed by EtOH/LPS stimulation for 30 min (for ROS levels), 6 h (for IL-6 mRNA), or 24 h (for IL-6 protein). The treatment of cells with ZnPP significantly suppressed the inhibitory effect of lycopene on intracellular (Figure 6A) and mitochondrial (Figure 6B) ROS, IL-6 mRNA (Figure 6C), and IL-6 (Figure 6D) levels, which had been increased by EtOH/LPS stimulation. Collectively, these results suggest that HO-1 contributes to the effect of lycopene in reducing the levels of ROS and IL-6 through the upregulation of HO-1 in EtOH/LPS-stimulated AR42J cells.

### 3.6. ML385 and ZnPP Inhibit the Effect of Lycopene on EtOH/LPS-Induced Mitochondrial Dysfunction in AR42J Cells

**Figure 7:**
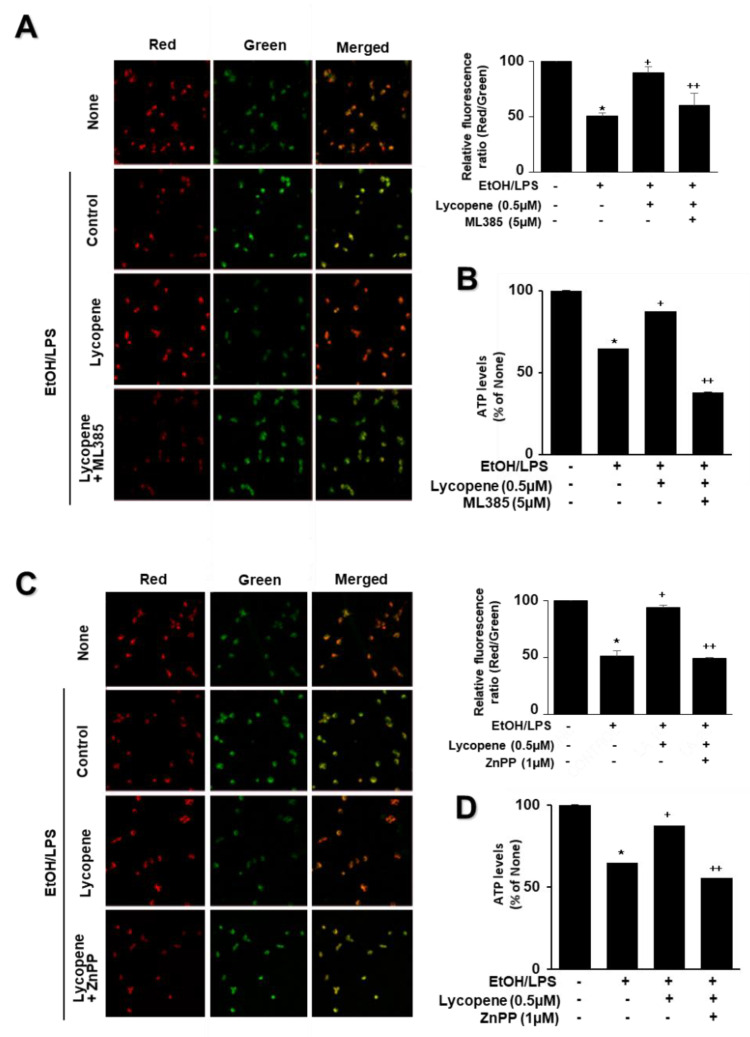
Effect of ML385 and ZnPP on mitochondrial dysfunction in ethanol (EtOH)/lipopolysaccharide (LPS)-stimulated and lycopene-treated AR42J cells. Cells (2 × 10^5^/2 mL/well) were pretreated with ML385 (5 μM) or ZnPP (1 μM) in the presence of lycopene (0.5 μM) for 1 h and stimulated with EtOH (at a final concentration of 250 mM)/LPS (10 μg/mL) for 30 min. (**A**,**C**) The cells were stained with JC-1 dye and visualized with a confocal laser scanning microscope (left panel). The MMP was determined by measuring the intensity of red emission relative to the intensity of green emission (right panel). A decrease in the red/green fluorescence intensity ratio indicates mitochondrial depolarization. Data are expressed as the mean ± SE (*n* = 3 in each group). (**B**,**D**) ATP levels were quantified using luminescent ATP substrate. The ATP concentration was determined by interpolating within the ATP standard reference. The fluorescence ratio and/or ATP level in control cells (cells treated without EtOH/LPS, lycopene, ML385, and ZnPP) was set as 100%. Data are expressed as the mean ± SE (*n* = 12 in each group). * *p* < 0.05 vs. control cells (cells without EtOH/LPS stimulation and without any treatment); + *p* < 0.05 vs. cells with EtOH/LPS stimulation and without any treatment;. ++ *p* < 0.05 vs. cells with EtOH/LPS stimulation and lycopene treatment. The cells incubated with tetrahydrofuran (THF, 0.01%) alone served as a vehicle control. For each experiment, lycopene-untreated cells received a vehicle THF (0.01%) alone instead of lycopene. ML385 and ZnPP were dissolved in dimethyl sulfoxide (DMSO). For each experiment, ML385- or ZnPP-untreated cells received a vehicle DMSO (0.05%). +, treatment.

To assess the involvement of Nrf2 and HO-1 on the effect of lycopene on EtOH/LPS-induced mitochondrial dysfunction, the cells were pretreated with ML385 (5 µM) or ZnPP (1 µM) with lycopene for 1 h and treated with EtOH/LPS for 30 min. As shown in Figure 7, lycopene prevented reduction in MMP and ATP levels in EtOH/LPS-stimulated cells, which was suppressed by ML385 and ZnPP. Therefore, inhibitory effect of lycopene on mitochondrial dysfunction may be mediated with Nrf2/HO-1 pathway in EtOH/LPS-stimulated cells.

## 4. Discussion

Heavy alcohol consumption is a potential risk factor for the development of pancreatitis, a painful and potentially fatal condition. Approximately one-third of acute pancreatitis cases are associated with alcohol intake, and 60–90% of patients with pancreatitis are heavy drinkers. It has been suggested that drinking more than 80 g, or approximately ten standard drinks, of alcohol per day, for a minimum of 6–12 years, is a risk factor for symptomatic pancreatitis [55]. Alcohol promotes the production of ROS while lowering cellular antioxidant levels, leading to oxidative stress in the pancreas [56]. Ample evidence indicates that oxidative stress plays an important role in the development of alcoholic pancreatitis. Excessive intracellular ROS levels upregulate the expression of the inflammatory cytokine IL-6, which contributes to acute pancreatitis [57]. We previously reported that EtOH and a palmitoleic acid cocktail activated NADPH oxidase to produce ROS and induce mitochondrial dysfunction and cell death in AR42J cells [58]. Furthermore, fatty acid ethyl esters cause mitochondrial dysfunction with reduced MMP, and impair the production of ATP in the pancreas [59]. Therefore, reducing ROS levels may be beneficial in reducing the risk of alcoholic pancreatitis.

Despite several trials, the pathogenesis of alcoholic pancreatitis remains elusive. This is because it is difficult to access pancreatic tissue in live human subjects, owing to its position in the abdomen. This can also be attributed to the lack of a suitable animal model for alcoholic pancreatitis research. Nevertheless, most recent studies have focused on the toxic effects of alcohol on acinar cells, and significant progress has been made. The question of why clinically overt pancreatitis develops in only a small number of alcohol abusers—approximately 5% of alcoholics—has evoked interest in identifying other predisposing factors besides alcohol that may confer individual susceptibility to the disease [60]. In response to this question, endotoxins, such as LPS, are candidate trigger factors for alcoholic pancreatitis, as patients with pancreatitis have increased serum LPS concentrations, which have been correlated with the severity of acute pancreatitis [61]. LPS-induced endotoxemia is relevant not only for alcoholic pancreatitis, but also for other metabolic diseases, such as diabetes and cardiovascular disease [62]. Several experimental models have revealed that LPS exacerbates the development of alcoholic acute pancreatitis, and continual exposure to LPS induces fibrogenesis and chronic pancreatitis [63,64,65,66]. In addition, LPS exerts a direct effect on pancreatic acinar cells. A single injection of LPS (10 ug/mL) into the superior pancreaticoduodenal artery of rabbits resulted in acute necrotizing pancreatitis. In human monocytes, LPS activates a series of intracellular signaling pathways that include the NF-κB and mitogen-activated protein kinase pathways. These signaling pathways, in turn, activate a range of transcription factors, such as NF-κB and AP-1, which coordinate the induction of inflammatory cytokines, including IL-6 [67]. Furthermore, HO-1 can inhibit the activation of NF-κB, which is associated with the pathogenesis of acute pancreatitis [68].

Lycopene, a carotenoid with 11 conjugated double bonds, has been shown to upregulate Nrf2-mediated HO-1 expression, leading to the inhibition of the transcription and nuclear translocation of NF-κB [69]. Lian et al. showed that enzymatic metabolites of lycopene induce Nrf2-mediated expression of antioxidant enzymes in human bronchial epithelial cells [70]. Serum levels of LPS are higher in excessive drinkers than those in non-drinkers. Therefore, we used EtOH/LPS-treated pancreatic acinar cells as an in vitro model of alcoholic pancreatitis.

This study was conducted to gain insight into the mechanism by which lycopene attenuates EtOH/LPS-induced increases in oxidative stress and levels of the inflammatory cytokine IL-6. Our results indicate that lycopene upregulates NQO1 and HO-1 expression via the activation of Nrf2, thereby alleviating the EtOH/LPS-stimulated increase in ROS and IL-6 expression. In addition, we demonstrated the inhibitory effect of lycopene on EtOH/LPS-induced mitochondrial dysfunction by blocking EtOH/LPS-induced decrease in MMP and ATP levels. ML385 is *N*-[4-[2,3-Dihydro-1-(2-methylbenzoyl)-1*H*-indol-5-yl]-5-methyl-2-thiazolyl]-1,3-benzodioxole-5-acetamide. It is a specific Nrf2 inhibitor that interferes with the binding of Nrf2 to the ARE sequence, as well as abolishes the inhibitory effect of lycopene on EtOH/LPS-induced reductions in NQO1 and HO-1 levels, increases in ROS and IL-6 levels, and mitochondrial dysfunction, in pancreatic acinar cells.

Moreover, we validated that ZnPP, an HO-1 inhibitor, reversed the inhibitory effect of lycopene on EtOH/LPS-induced increases in ROS and IL-6 levels, as well as mitochondrial dysfunction, in AR42J cells. These results show that Nrf2-mediated expression of the antioxidant enzyme HO-1 is associated with the protective role of lycopene in an in vitro alcoholic pancreatitis model. Lin et al. showed that the inhibitory effect of lycopene on LPS-induced expression of cyclooxygenase-2 is mediated by HO-1 activation in microglial cells [71]. Lycopene alleviates hepatic hypoxia/reoxygenation injury by promoting the translocation of Nrf2 into the nucleus, activating the Nrf2/HO-1 pathway in hepatic cells [72].

Regarding the possible toxicity of THF, we used 0.01% of THF for a vehicle of lycopene. Even though THF is a toxic compound, THF (0.1%) did not decrease the cell viability of prostate cancer cell line DU145 and SH-SY5Y Neuroblastoma cell line SY5Y [73,74]. In addition, THF, up to the maximum concentration of 1.25% in the cell culture medium, did not affect the viability of the gastric cancer cell line HepG2 [75]. Therefore, 0.01% THF, used as control in the present study, may not affect cell viability.

## 5. Conclusions

Lycopene enhances antioxidant defense activities via the upregulation of Nrf2 signaling, and its target antioxidant genes NQO1 and HO-1, in pancreatic acinar cells. Reducing ROS levels using lycopene suppresses the EtOH/LPS-induced expression of IL-6, increases in intracellular and mitochondrial ROS levels, and mitochondrial dysfunction, in pancreatic acinar cells.

Therefore, the consumption of lycopene-rich foods may prevent the development of alcohol/LPS-associated pancreatitis by activating Nrf2-mediated expression of NQO1 and HO-1, thereby downregulating ROS-mediated IL-6 expression in pancreatic acinar cells.

## Data Availability

The data used to support the findings of this study are included within the article.

## Abbreviations

CD14: cluster of differentiation 14; CYP2E1: cytochrome P4502E1; DCF: dichlorofluorescein; DMSO: dimethyl sulfoxide; EDTA: ethylenediaminetetraacetic acid; EtOH: ethanol; FAEE: fatty acid ethyl ester; Keap1: Kelch-like ECH1-associated protein 1; HO-1: heme oxygenase-1; LPS: lipopolysaccharides; MD-2: myeloid differentiation factor-2; MMP: mitochondrial membrane potential; NF-κB: Nuclear factor-κB; NQO1: NAD(P)H quinone oxidoreductase 1; Nrf2: nuclear erythroid-2-related factor 2; PBS, phosphate-buffered saline; ROS: reactive oxygen species; SDS: sodium dodecyl sulfate; THF: tetrahydrofuran; TLR4: toll-like receptor 4; ZnPP: Zinc protoporphyrin.
